# Inherited *FGFR2* mutation in a Chinese patient with Crouzon syndrome and luxation of bulbus oculi provoked by trauma: a case report

**DOI:** 10.1186/s12886-019-1217-8

**Published:** 2019-10-22

**Authors:** Ji Yang, Tao Tao, Hai Liu, Zhu-Lin Hu

**Affiliations:** 1 Department of Ophthalmology, Second people’s hospital of Yunnan province, Kunming, 650000 China; 2 The eye disease clinical medical research center of Yunnan province, Kunming, 650000 China; 3 The eye disease clinical medical center of Yunnan province, Kunming, 650000 China

**Keywords:** Crouzon syndrome, Luxation of eyeball, Mutation screening, *FGFR2*

## Abstract

**Background:**

Crouzon syndrome (CS), which results from fibroblast growth factor receptor 2 mutations, is associated with craniosynostosis, exophthalmos, and other symptoms. Herein, we report the genetic abnormalities detected in a Chinese family with autosomal dominant CS, combined with luxation of the eyeball. This luxation was a consequence of the trauma to the shallow orbits.

**Case presentation:**

The proband was a 4-year-old boy. He accidentally fell, following which luxation of the bulbus oculi occurred immediately. Computed tomography and magnetic resonance imaging clearly revealed ocular proptosis. Upon physical examination, the proband, his father, and grandfather had ocular proptosis, shallow orbits, and mid-face hypoplasia. However, their hands and feet were clinically normal. Genomic DNA was extracted from the peripheral blood through a polymerase chain reaction performed for the target sequence. Genetic assessments revealed a heterozygous missense mutation (c.1012G > C, p.G338R) in exon 10 of the human *FGFR2*, cosegregated with the disease phenotype in this family. These findings confirmed the diagnosis of CS.

**Discussion:**

CS is usually caused by *FGFR2* mutations. While there are a few reports of luxation of the bulbus oculi in Chinese families with CS, the ocular proptosis, shallow orbits, combined with luxation of eyeball after trauma observed in this patient were particularly interesting. Our findings enhance the current knowledge of traumatic luxation concomitant with CS.

## Background

Crouzon syndrome (CS), caused by fibroblast growth factor receptor 2 (FGFR2) mutations, is associated with craniosynostosis. The incidence is approximately 16.5/1,000,000 [1]. The common manifestations of CS include brachycephaly, exophthalmos, divergent strabismus to ocular hypertelorism, and mandibular prognathism. Heterozygous mutations of *FGFR2*, located at 10q26, have been identified in autosomal dominant CS. The FGFR2 protein consists of three extracellular immunoglobulin-like (Ig) domains (Ig I, Ig II, and Ig III), a single transmembrane segment, and a cytoplasmic tyrosine kinase domain [2]. To the best of our knowledge, nearly 60 *FGFR2* mutations have been identified in association with CS. Meanwhile, approximately 95% of mutations in CS occur in exon 8 and exon 10 of the *FGFR2* gene. Each of these exons separately codes for the Ig IIIa (8) and Ig IIIc (10) domains [3]. We report the genetic abnormalities in a Chinese family with autosomal dominant CS combined with luxation of the eyeball in this study. It is noteworthy that the luxation in our patient was a sequela of trauma to the shallow orbits.

## Case presentation

**Fig. 1:**
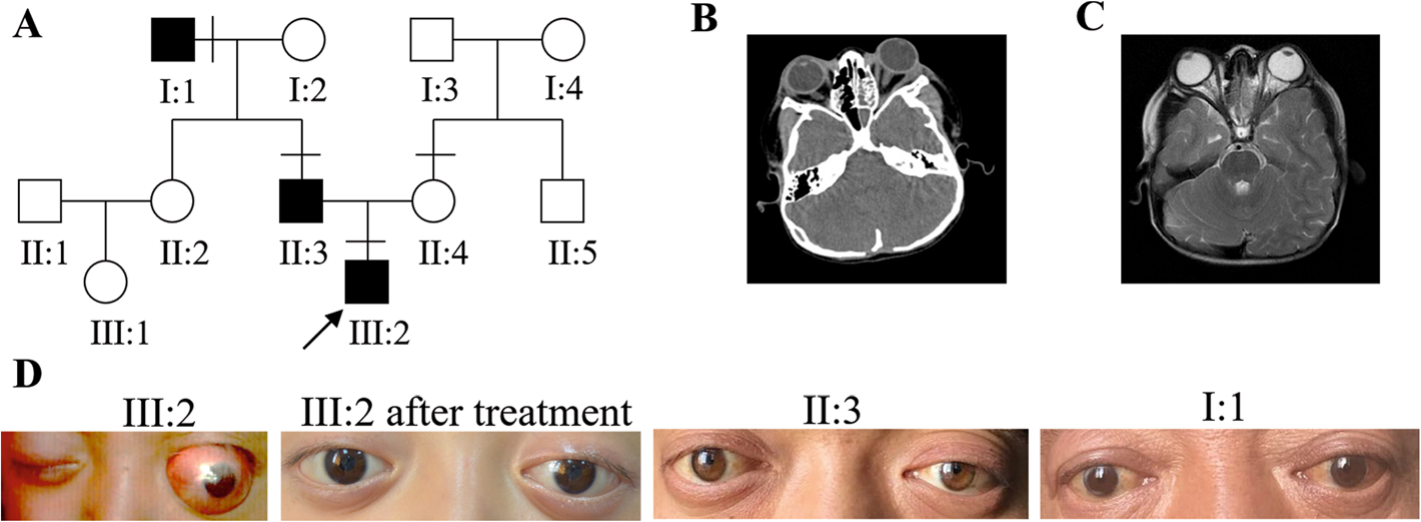
Patients with Crouzon syndrome and clinical examination of the proband. **a** Three-generational Crouzon syndrome pedigree. Affected individuals are indicated by filled symbols and the proband is marked with an arrow. **b**, **c** Computed tomography and magnetic resonance imaging did not reveal retrobulbar hematoma and revealed shallow orbits and obvious ocular proptosis in the proband. **d** Facial photographs of patients with Crouzon syndrome

A pedigree with CS was recruited for this study (Fig. 1a). The patient received a systemic evaluation, and we collected peripheral blood samples from the patient and his lineal relatives. This study adhered to the tenets of the Declaration of Helsinki. The ethics committee of The Second People’s Hospital of Yunnan province approved the protocol, and we obtained written informed consent from all the study participants. Genomic DNA was extracted from the peripheral blood. Polymerase chain reaction (PCR) for the target sequence of *FGFR2* was performed, according to the methods described previously [4].

The proband was a 4-year-old boy. He fell accidentally, following which luxation of the bulbus oculi occurred immediately (Fig. 1d). The patient’s family brought him to our hospital for treatment. We observed that the patient’s left eyeball was clearly dislocated and accompanied by hypophasis of the left eye. The eye movements were restricted in all directions. External strabismus was also observed. The relative afferent pupillary defect (RAPD) was negative. Upon physical examination, the proband (III:2), his father (II:3, 28 years old), and his grandfather (I:1, 54 years old) had ocular proptosis, shallow orbits, and mid-face hypoplasia, but clinically normal hands and feet (Fig. 1d). The boy’s father and grandfather had normal vision, while displaying a ‘surprised look’. The best corrected visual acuity (BCVA) of individual III:2 (4 years old) was 0.5 for the right eye, and 0.1 for the luxated left eye. The visual acuity of the left eye was in a precarious condition due to severe exposure keratitis and traumatic dislocation of the eyeball. The orbital pressure in left orbit was higher than that in the right orbit. Computed tomography and magnetic resonance imaging did not reveal retrobulbar hematoma and revealed shallow orbits and ocular proptosis in patient III:2 (Fig. 1b, c). Therefore, mannitol (10 g, q8h, intravenous drip) was administered for symptomatic reduction of orbital pressure for 3 days. At the same time, tobramycin eye ointment (topical instillation, qn) and carbomer eye drops (topical instillation, q8h) were administered to the left eye for 3 days to protect it from exposure keratitis and obstinate conjunctivitis. After treatment, the dislocated bulbus oculi reverted back to the orbit and left eyelid could be closed. The BCVA of the left eye increased to 0.2 (Fig. 1d). The restriction in eye movement improved after treatment. However, mild restricted internal eye movement and mild external strabismus were observed. The patient was re-examined at our hospital 1 week after discharge. There was no obvious dislocation of the eyeball. The BCVA of left eye was maintained at 0.2. Subsequently, the patient was lost to follow-up.

**Fig. 2:**
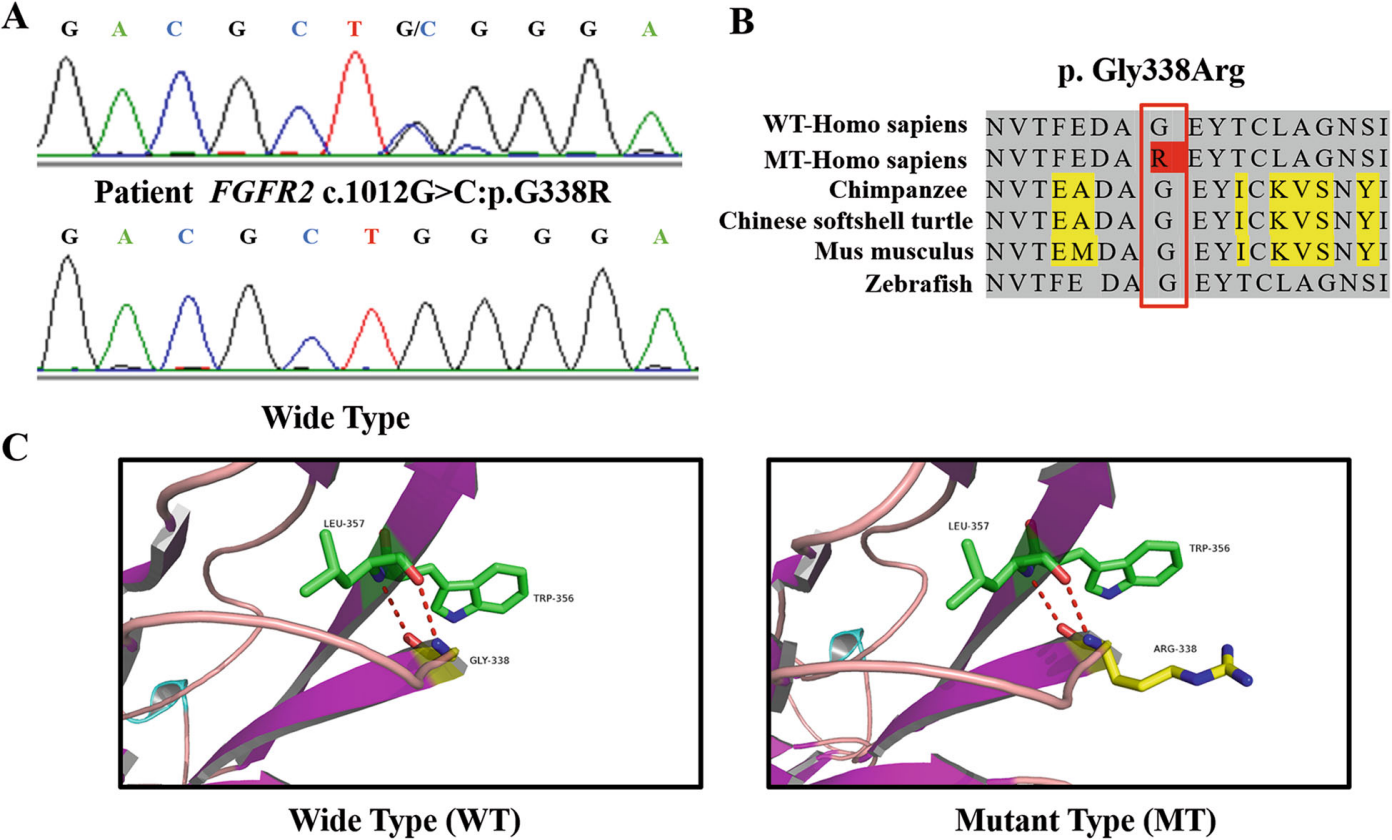
Identification of mutations in the family. **a** Sequencing chromatograms of the heterozygous missense mutation (c.1012G > C, p.G338R) in exon 10 of the *FGFR2* (upper) and the wild-type sequence (below). **b** Amino acid sequence of *FGFR2* around G338R in a family with Crouzon syndrome, which is a highly conserved segment of the FGFR2 protein in humans and other species. **c** Molecular modelling of the wild-type and mutant FGFR2 protein

Genetic assessments revealed a heterozygous missense mutation (c.1012G > C, p.G338R) in exon 10 of the human *FGFR2*, cosegregated with the disease phenotype in this family (Fig. 2a). This mutation resulted in the replacement of hydrophilic glycine with more hydrophilic arginine at codon 338 (p. Gly338Arg) at the immunoglobulin (Ig)-like domain 3. Moreover, the missense mutation is not found in normal individuals, including II:4, who was the proband’s mother.

To study the structure of the mutant FGFR2 protein, three-dimensional crystal structures of the wild-type and mutant FGFR2 (residues 1–520) were constructed based on the human FGFR2, with a sequence confidence of 100% and coverage of 59%. Structural modeling of the FGFR2 protein (Fig. 2c) demonstrated that G338R was located in the β-sheet, which is required for receptor binding [5]. The mutated area was found in a highly conserved segment of the FGFR2 protein in humans and other species (Fig. 2b). We considered the variation as ‘likely pathogenic’ according to the standards and guidelines from American College of Medical Genetics and Genomics (ACMG), and 4 bioinformatics tools (SIFT, Mutation Taster, Polyphen2, REVEL) [6].

## Discussion

CS is usually caused by *FGFR2* mutations and accounts for 4.8% of cases with craniosynostoses. CS is easily diagnosed when patients present with abnormal facial structures, especially exophthalmos and maxillary or mandibular prognathism, which can be visualized on fetal ultrasonography performed at 35 weeks of gestation, for orbital growth retardation [7]. For treatment, surgical intervention for the malformation of the skull in CS is recommended, in the presence of orbital proptosis and maxillary or mandibular prognathism [8]. Our patient presented with luxation of the bulbus oculi and hypophasis of the left eye. The treatment aimed to reduce orbital pressure and protect the affected eye from exposure keratitis and obstinate conjunctivitis. The symptoms were relieved and the BCVA increased after treatment.

In the present study, we reported the identification of a missense mutation (c.1012G > C, p.G338R) in a Chinese family with autosomal dominant CS. A few *FGFR2* mutations have been found to affect osteoblast and chondrocyte cell proliferation in vitro [9]. At the same time, a minor change in *FGFR2* can inevitably lead to primordial bone changes [10]. Furthermore, mutant-type *FGFR2* (c.1012G > C) can affect craniofacial growth, by increasing alkaline phosphatase gene and osteocalcin gene expression, inevitably leading to CS [11]. The clinical characteristics of our patient supported a diagnosis of CS, while there are a few reports of luxation of the bulbus oculi in Chinese families with CS. It is of interest that our patient had ocular proptosis and shallow orbits, combined with luxation of the eyeball after trauma.

In conclusion, we identified a mutation in *FGFR2* that caused CS [11, 12]. Edema of the periorbital tissue is observed in the event of blunt injuries to the forehead and periorbital area. The shallow orbits observed in patients with CS patients have limited space to accommodate edematous tissue. Therefore, these patients are more likely to develop eyeball luxation. Our findings enhance the current knowledge of CS concomitant with traumatic luxation of the eyeball.

## Data Availability

All data generated or analyzed in this report are included in this published article.

## Abbreviations

CS: Crouzon syndrome
FGFR2: Fibroblast growth factor receptor 2
